# Examining the Association Between Serotonergic Antidepressants and Blood Transfusion Requirements in Orthopaedic Surgery: A Comprehensive Analysis

**DOI:** 10.7759/cureus.45988

**Published:** 2023-09-26

**Authors:** Faiez Iqbal, Aneemaa Narayan, Mallicka Chatrath, Mohammad Iqbal

**Affiliations:** 1 Orthopaedics and Trauma, Kota Trauma Hospital, Kota, IND

**Keywords:** serotonergic drug, patient-care, trauma and orthopedic surgery, hemoglobin level, blood transfusion safety

## Abstract

Aim: The study aimed to investigate the association between the use of serotonergic antidepressants and blood transfusion requirements in orthopedic surgical patients.

Methodology: A retrospective follow-up study was conducted at Kota Trauma Hospital, Kota, Rajasthan focusing on patients who underwent orthopedic surgeries between November 2021 through December 2022. Patients were categorized into two groups: users of serotonergic antidepressants and users of other antidepressants (non-selective serotonergic antidepressants). The requirement for blood transfusion for both groups was assessed. Covariate factors, such as medication use and comorbidities (e.g., diabetes mellitus, cardiovascular diseases), were examined to control potential confounding variables.

Result: A total of 170 subjects with complete medical records were included in the study. The results presented a significant association between the usage of serotonergic antidepressants and blood transfusion requirements (p=0.001). While no significant differences between the two groups were observed in perioperative hemoglobin levels and fluid infusion, there was a significant difference in blood loss and postoperative drainage.

Conclusion: Serotonergic antidepressant usage was correlated with increased blood transfusion requirements in orthopedic surgery patients on antidepressants. The study underscores the importance of considering medication factors in perioperative management and highlights potential implications for patient care strategies.

## Introduction

Selective serotonin reuptake inhibitors (SSRIs) are extensively used pharmaceuticals for the treatment of depression, owing to their notable effectiveness and favorable safety characteristics [1,2]. Over the last two decades, there has been a significant rise in the use of antidepressant medications, with SSRIs progressively replacing tricyclic agents as the preferred first treatment option. These antidepressants have gained significant popularity due to their reduced toxicity and improved tolerability [3,4]. There is an observed correlation between the administration of antidepressant drugs that possess serotonergic action and an increased propensity for bleeding [5]. The probable reason for poor hemostasis is the depletion of serotonin from platelets due to the suppression of serotonin reuptake by SSRIs. This is because serotonin plays a role in promoting platelet aggregation [6]. The incidence of bleeding events associated with SSRIs seems to be directly correlated with the extent of serotonin reuptake inhibition [7].

A number of case reports and observational studies have shown a potential association between SSRIs and an increased risk of bleeding [8,9]. The pharmacological mechanism responsible for this possible detrimental impact has been thoroughly elucidated. The serotonin release in platelets plays a crucial role in their aggregation process [10]. Platelets lack the ability to engage in serotonin synthesis, instead relying on serotonin transporters to uptake serotonin from the bloodstream [11]. SSRIs exert their pharmacological effects by inhibiting the reuptake of serotonin transporters, hence reducing the concentration of serotonin inside platelets during a prolonged course of therapy [12].

A number of observational studies have been conducted to investigate the association between the risk of bleeding and the use of SSRIs. However, it is worth noting that only two studies [13,14] have specifically focused on examining the relationship between SSRIs and bleeding risk in the perioperative period. Patients who were on SSRIs had a higher magnitude of blood loss and thus required blood transfusions more often throughout Orthopaedic surgical procedures. Conflicting evidence has been found about the potential influence of perioperative usage of SSRIs on hemostasis impairment. Therefore, given the restricted nature of the available data, the potential impact of SSRIs on the risk of perioperative bleeding remains a significant therapeutic consideration. The potential impact of SSRIs on hemostasis and subsequent bleeding in patients is a crucial consideration for anaesthesiologists. However, the existing body of information regarding the perioperative treatment of SSRIs is limited, particularly in terms of determining whether these medications should be continued or discontinued [15,16].

The study conducted by Movig et al. [17] investigated the potential correlation between the use of SSRIs and the need for blood transfusions in individuals who had several orthopaedic surgical interventions. The incidence of blood transfusion was almost four times higher in the group of individuals using SSRIs compared to those who did not use SSRIs. The aforementioned link was corroborated in further research conducted by van Haelst et al. in 2010 [18]. The clinical relevance of the findings remains questionable due to the limited number of cases that had significant perioperative blood loss. Thus, the objective of this research was to investigate the correlation between the perioperative administration of SSRIs and the volume of blood loss during surgical procedures, as well as the need for perioperative transfusions.

## Materials and methods

Study participants

The research was carried out at Kota Trauma Hospital, Kota, Rajasthan. Patients who had elbow, knee, or shoulder implant surgeries between January 1, 2021, and December 31, 2022, were included in the research. Patients' data were gathered from hospital records. The Medical Ethics Committee approved the study plan, and patients were requested to provide informed permission.

Study design

A retrospective study design was used for the current study. Patients were excluded if they did not provide informed consent, their medical records were missing or incomplete or had incomplete drug prescription data. The primary outcome variable was the necessity for blood transfusions during surgery. Secondary outcomes included bleeding during the procedure, the need for infusions, and the loss of drainage fluid. Prescription data were received from neighborhood pharmacies, and additional pre and postoperative information was retrieved from hospital records.

Covariate factors

The study examined potential factors that could influence the results, including the use of medications like corticosteroids, calcium-channel blockers, Fe supplements, aspirin, non-steroidal anti-inflammatory drugs (NSAIDs), and vitamin K (antagonists). Additionally, concomitant diseases such as diabetes mellitus, renal diseases, bleeding ulcers, and cardiovascular diseases were taken into account as potential factors that could confound the association being studied.

Statistical analysis

Statistical Package for Social Sciences (SPSS) version 25 (IBM Corp., Armonk, NY, USA) was used to perform the statistical tests. The mean and standard deviation were used to describe all continuous values, whereas numbers and percentages were used to represent categorical data. Descriptive statistics were used to determine the frequencies and proportions. Inferential statistics were used to determine the association between variables and to compare the data. The χ2 test was used to determine the varied proportions of categorical variables.

## Results

A total of 170 patients were included in the study. Table 1 provides an overview of the general characteristics of the study patients, with a distinction made between those who required blood transfusion during surgery (N=78) and those who did not (N=92). In terms of age distribution, a slightly higher percentage of patients over the age of 60 underwent blood transfusion (53) compared to the no-blood transfusion group (57). Gender distribution showed that a larger proportion of females underwent both transfusion (48) and non-transfusion (58) procedures. Preoperative hemoglobin levels were lower in the blood transfusion group (1.47 g/dl) compared to the no-blood-transfusion group (1.68 g/dl). Regarding the type of implant used, a higher percentage of patients who underwent elbow implant surgery required blood transfusion (60%) compared to knee (13%) and shoulder (7%) implant surgeries. In terms of anesthesia, both groups were relatively evenly split between general and spinal anesthesia.

**Table 1:** Overview of Study Patients' General Characteristics, Including Subgroups Based on Blood Transfusion Requirement During Surgery g/dl: Grams per Deciliter; NSAIDs: Non-Steroidal Anti-Inflammatory Drugs; Fe: Iron

Characteristics	Blood transfusion (N=78)	No blood transfusion (N=92)
Age		
<60	25	35
>60	53	57
Gender		
Male	30	34
Female	48	58
Preoperative haemoglobin (g/dl)	1.47	1.68
Type of implant (%)		
Elbow	60	65
Knee	13	22
Shoulder	7	5
Anaesthesia (%)		
General	56	67
Spinal	22	25
Medication (%)		
Clomipramine hydrochloride	6	4
Paroxetine	3	5
Venlafaxine hydrochloride	1	3.5
Amitriptyline	1	13
Imipramine	2	10
Phenelzine	0	5
Corticosteroids	2	4
Calcium channel blockers	10	10
Fe supplements	7	3
Aspirin	10	13
NSAIDs	47	35
Vitamin K (antagonists)	2	1
Comorbidity (%)		
Diabetes mellitus	8	9
Renal diseases	0	5
Bleeding ulcers	0	10
Cardiovascular diseases	3	3

The usage of various medications was recorded, and it was noted that certain antidepressants (e.g., amitriptyline, imipramine) were more commonly used in the blood transfusion group compared to the no-blood-transfusion group. NSAIDs were more prevalent among patients who did not require a blood transfusion.

Morbidity and comorbidity percentages were also assessed. While diabetes mellitus, cardiovascular diseases, and renal diseases were relatively evenly distributed between the two groups, bleeding ulcers were notably higher in the no-blood-transfusion group (10%).

Table 2 illustrates a comparison of hematologic measures between patients using serotonergic antidepressants and those not using serotonergic antidepressants. In the group using serotonergic antidepressants, 23% of patients required blood transfusion, while only 4% did in the non-serotonergic antidepressant group, with a statistically significant p-value of 0.001.

**Table 2:** Comparison of Hematologic Measures Between Patients not Using Serotonergic Antidepressants and Patients Using Serotonergic Antidepressants SD: Standard deviation; p-value: Probability Value<0.05, 0.01, 0.001 denotes statistical significance

	Serotonergic antidepressants (n=78)	Non-serotonergic antidepressants (n=92)	P value
Blood transfusion (%)	23	4	0.001
Perioperative haemoglobin (Mean±SD)	10.56±1.16	10.62±1.24	0.07
Perioperative blood loss (Range)	0-7500	0-6500	0.001
Perioperative fluid infusion (Range)	300-5000	200-9500	.24
Postoperative drainage (Range)	60-1750	0-3000	0.05

Regarding hematologic parameters, the perioperative hemoglobin levels showed no substantial difference between the two groups, with mean levels of 10.56±1.16 g/dl for serotonergic users and 10.62±1.24 g/dl for non-serotonergic users, resulting in a p-value of 0.07. However, the perioperative blood loss was notably higher in the serotonergic antidepressant group (0-7500 ml) compared to the non-serotonergic group (0-6500 ml), with a significant p-value of 0.001.

Perioperative fluid infusion ranged from 300 ml to 5000 ml for serotonergic users and 200 ml to 9500 ml for non-serotonergic users, with no significant difference (p=0.24). Postoperative drainage ranged from 60 ml to 1750 ml for serotonergic users and 0 ml to 3000 ml for non-serotonergic users, resulting in a p-value of 0.05, indicating a moderate difference.

Table 3 presents the odds ratios (OR) for blood transfusion in relation to age, sex, comedication, and comorbidity factors. For the age group above 60, the odds ratio was 0.89, indicating a relatively neutral association with blood transfusion. Gender-wise, females exhibited an odds ratio of 0.79, suggesting no significant influence on blood transfusion likelihood. Patients on serotonergic antidepressants have an odds ratio of 2.51, potentially indicating an increased risk of blood transfusion. Non-serotonergic users displayed an odds ratio of 0.72, suggesting a wide range of possibilities. The use of Fe supplements and NSAIDs showed odds ratios of 1.99 and 1.74 respectively, indicating the increased risk of perioperative blood transfusion. The increased risk was not found in non-serotonergic antidepressants, corticosteroids, or calcium-channel blockers and vitamin K antagonist treatment. Comorbidity factors yield odds ratios within specific ranges. Diabetes mellitus showcased an odds ratio of 1.03, implying a range of effects on blood transfusion probability. Cardiovascular diseases demonstrated an odds ratio of 1.42, suggesting potential varied impacts.

**Table 3:** Odd ratios for the requirement of blood transfusion according to several demographic factors OR: Odds Ratio; CI: Confidence Interval; Fe: Iron; NSAIDs: Non-Steroidal Anti-Inflammatory Drugs

Characteristics	OR (95% CI)
Age	
>60	0.89(0.67-1.61)
Gender	
Female	0.79(0.48-1.52)
Medication	
Serotonergic	2.51(1.35-6.55)
Non-serotonergic	0.72(0.10-5.95)
Corticosteroids	0.51(0.06-2.43)
Calcium-channel blockers	1.10(0.48-2.63)
Fe supplements	1.99(0.51-7.85)
Aspirin	0.82(0.32-2.06)
NSAIDs	1.74(0.92-2.85)
Vitamin K (antagonists)	1.02(0.12-9.54)
Comorbidity	
Diabetes mellitus	1.03(0.34-2.81)
Cardiovascular diseases	1.42(0.27-5.74)

## Discussion

This retrospective research examined the correlation between the perioperative administration of serotonergic antidepressants and the likelihood of receiving a blood transfusion of two or more units in patients who had orthopaedic surgery. The findings revealed a threefold elevation in the risk of such blood transfusions among those who were exposed to serotonergic antidepressants during the perioperative period. The results of the current research indicated that those who were prescribed serotonergic antidepressants had a statistically significant increase in blood loss during orthopaedic surgical procedures compared to those who were not taking any antidepressant medication. The available case reports have provided evidence suggesting a potential association between serotonergic antidepressants and complications linked to bleeding. Previous research has shown that the use of serotonergic antidepressants is significantly linked to an increased likelihood of experiencing serious gastrointestinal bleeding, especially in those who are concurrently using NSAIDs. In a recent study conducted by Yang et al. (2023) [19], it was shown that there was a threefold increase in the risk of receiving two or more units of packed red blood cells (PRBCs) transfusion. Comprehensive incidence research conducted an estimation of the absolute risk associated with serotonergic antidepressant use. The findings indicated a significant positive association between the total number of blood transfusions administered and the duration of hospitalization.

Orthopaedic and rheumatology patients who suffer from excruciating osteoarthritis often take NSAIDs before undergoing joint replacement surgery. According to Friedman et al. (2013) [20], NSAIDs increase the risk of perioperative blood loss in patients having total knee arthroplasty. Our most recent NSAID research supports these studies.

Patients on calcium-channel blockers did not have a higher risk of perioperative blood transfusion in the current trial, but they did need more post-operative blood transfusions. Rheumatoid arthritis is often treated in ambulatory people who receive intramuscular methotrexate. The requirement for perioperative blood transfusions in orthopaedic patients with rheumatoid arthritis is supported by our data even though methotrexate itself is not known to be linked to a higher risk of bleeding [17,21].

A reduction in intraplatelet serotonin concentrations that impact platelet aggregation may be the underlying cause of the SSRIs' increased risk of bleeding [22]. The surgery itself has been linked to a reduction in platelet serotonin and a corresponding rise in plasma serotonin [23]. In surgical patients using SSRIs, the interaction of the two effects may increase the risk of bleeding since it may decrease hemostasis [24]. In contrast, the study conducted by Andreasen et al. [25] revealed a lack of significant need for blood transfusions in patients who were prescribed SSRIs prior to undergoing coronary artery bypass grafting.

Our investigation may have some limitations regarding the influences of variables (other than the exposure of interest) on the risk of the outcome, similar to previous observational studies. But, we think it is unlikely that any possible biases have had a big impact on our findings. First, information bias seems to be very improbable given that serotonergic antidepressants are not often related to bleeding issues during orthopaedic surgeries in routine clinical practice. Second, we think that selection bias was not a significant problem in this research since a well-defined population was used to sample all of the patients. Recall bias is minimal since all data were gathered in the past according to the study's retrospective design. The hematological laboratory, which has highly secure procedures for managing and recording blood transfusion needs in the clinic, provided the data required for end-point definition electronically.

The study has some limitations including sample size and study area. As the research only included one hospital of a city, the results of a larger sample size may vary.

## Conclusions

The current research revealed a significant association between the usage of serotonergic antidepressants and blood transfusion requirements. Due to the common and rising use of antidepressants, the link between serotonergic antidepressants and perioperative blood transfusion in orthopaedic surgery may represent a risk to the health of older patients. There is a chance that a lot of patients will be exposed to this danger. The study underscores the importance of considering medication factors in perioperative management and highlights potential implications for patient care strategies.
